# Reactivity and Corrosion Behaviors of Ti6Al4V Alloy Implant Biomaterial under Metabolic Perturbation Conditions in Physiological Solutions

**DOI:** 10.3390/ma14237404

**Published:** 2021-12-02

**Authors:** Lidia Benea, Nicoleta Simionescu-Bogatu

**Affiliations:** 1 Competences Center, Interfaces-Tribocorrosion-Electrochemical Systems, Dunarea de Jos University of Galati, 47 Domnească Street, RO-800008 Galati, Romania; 2 Faculty of Engineering, Dunarea de Jos University of Galati, 47 Domnească Street, RO-800008 Galati, Romania

**Keywords:** Ti6Al4V alloy, electrochemical characterization, inflammatory effect, corrosion

## Abstract

The corrosion of implant biomaterials is a well-known critical issue when they are in contact with biological fluids. Therefore, the reactivity of Ti6Al4V implant biomaterials is monitored during immersion in a Hanks’ physiological solution without and with added metabolic compounds, such as lactic acid, hydrogen peroxide, and a mixture of the two. Electrochemical characterization is done by measuring the open circuit potential and electrochemical impedance spectroscopy performed at different intervals of time. Electrochemical results were completed by morphological and compositional analyses as well as X-ray diffraction before and after immersion in these solutions. The results indicate a strong effect from the inflammatory product and the synergistic effect of the metabolic lactic acid and hydrogen peroxide inflammatory compound on the reactivity and corrosion resistance of an implant titanium alloy.

## 1. Introduction

Reactivity and corrosion of biomaterials are complex processes and their effects are very important because they could cause various pathological diseases or trauma in the biomedical field. These phenomena consist of the partial or complete destruction of metallic implants, caused by chemical, electrochemical, or biochemical reactions that take place between the metallic implants and certain factors from the body’s environment [1,2,3,4,5,6,7,8,9,10,11,12].

Over the last 20 years, titanium and its alloys have gained increasing attraction as materials used for surgical applications and bone replacements, due to their special mechanical, chemical, and biocompatibility properties [1]. It has been clearly established that titanium is biocompatible, inert, and immune to corrosion in contact with some fluids and tissues from the human body [2].

Titanium and its alloys are materials with corrosion resistance and good biocompatibility in contact with living tissue. The favorable biological response is due to the titanium oxide layer that forms spontaneously on its surface [3,4].

This oxide layer has a thickness of about 1–5 nm and can prevent the problems caused by the release of metal ions when the surface of titanium and titanium alloys are exposed to the bodily fluids of living organisms for a long time [5]. On the other hand, for the bone integration process, contact between the metal implant and the living tissue is made through the oxide layer on the implant’s surface [5].

Therefore, this layer of native oxides derived from the alloying elements of the alloy prevents the propagation of corrosion processes on the surface of the biomaterials. Interruption of the oxide film may be due to active oxygen species, proteins, cells, or organic ions present at this level; therefore, corrosion cycles can be initiated and the reactivity of biomaterial surface is activated. Implantation failure may be influenced by mobility, implant exposure in the oral cavity, and material wear [6,7]. Thus, the most important requirement for the long-term success of implants is a stable interface between the biomedical implant and the surrounding tissue [8].

The pathological processes of mucositis, such as peri-implantitis, may favor the rejection of the implant at the level of the dental arches and favor the accumulation of bacterial biofilms on the implant surfaces, thus initiating an increased number of inflammatory cells in the connective subepithelial tissue. In addition, saliva and bacteria flow under functional loading, creating an additional effect, along with the translocation of titanium particles into the peri-implant tissue and peri-implant bacteria into the gap of the implant–abutment connection. The presence of saliva, bacteria, and their subproducts, as well as the wear and onset of corrosion of the metal parts within the implant–abutment connection can lead to biological (mucositis and/or peri-implantitis) and mechanical (corrosion, screw loosening, fracture, and/or fatigue) implant failures. Saliva also plays an important role in the corrosion of dental implants [6,7,9,10,11,12,13]. The dental implant’s surface is constantly exposed to saliva via the gingival sulcus.

Saliva can act as a weak electrolyte, and the oral cavity can simulate an electrochemical cell that facilitates dissolution of the oxide layer. Further electrochemical corrosion of titanium alloy may lead to pitting corrosion and the eventual release of corrosion products. Factors that can affect the reactivity and corrosion resistance of the titanium alloy surface include inflammation of the surrounding tissue (which can produce local acidosis) and the acidic environment created by lactic acid released by bacteria [10,11,14].

An acidic environment, such as lactic acid, can affect the integrity of the titanium oxide layer because it depletes the oxygen sources needed for re-passivation, thus hindering the ability of the titanium surface to re-form the oxide layer [11]. The creation and use in laboratory tests of synthetic media either to monitor the degradation of biomaterials over time or to study the physical–chemical properties of biological fluids is essential in understanding the mechanisms that occur at the contact point between metallic implants and a living organism. The uses of a varied number of synthetic media used for the simulation of biological fluids and the monitoring of the corrosion behavior of different biocompatible materials can be found in the literature [15,16].

One of the best-known simulated environments used for reactivity and corrosion investigations of biomaterials is the Hanks’ solution [17,18,19]. Other solutions include Hanks’ with and without hydrogen peroxide (H_2_O_2_) [17]; Hanks’ solution with and without albumin [20]; Ringer’s solution [21]; Fusayama Meyer [22,23]; Fusayama Meyer with addition of albumin and hydrogen peroxide [24]; Fusayama Meyer with addition of fluoride ions, hydrogen peroxide, and lactic acid [25]; artificial saliva [26]; artificial saliva with lactic acid [27]; saline solution 0.9% NaCl [28]; 0.1 M NaCl with 0.1 M lactic acid [29]; and phosphate buffered saline solution [30].

However, there are very few studies reported in the literature that follow the reactivity and corrosion behavior during different immersion periods of Ti6Al4V alloy in Hanks’ solution with the addition of hydrogen peroxide that present in inflammatory situations and using lactic acid as metabolic product [25]. These compounds can affect the corrosion resistance of implant alloy.

Therefore, the mechanism of degradation remains unclear. Lactic acid is used in this study because it is a poor organic acid that plays an important role in biochemical processes. It circulates in the blood system in small amounts because it is produced by the muscles of the body during exercise to obtain energy and metabolizes glucose in the absence of oxygen. Lactic acid also may be present in the oral cavity and can also be found in food and beverages [31,32]. The reactive oxygen species, hydrogen peroxide (H_2_O_2_), is used in this study to simulate the inflammatory conditions that can occur in the human body [24]. The results presented in this study are useful for a better understanding the degradation process and the effects over time of the main compounds responsible for the reactivity and corrosion of titanium alloy (based implants) in the biological or oral environment, as simulated by, respectively, hydrogen peroxide and lactic acid that are added to Hanks’ solution.

The objective of this research paper is to study the effect of time and the addition of lactic acid, hydrogen peroxide, and these two mixed compounds on the reactivity and corrosion behavior of a Ti6Al4V titanium alloy as an implant material through electrochemical characterization. More precisely, this study brings us clearer information than other studies on the mechanism and kinetics of the degradation of titanium alloy implants in harsh conditions, including inflammation and the presence of lactic acid acting as a metabolic product.

The experimental results show a slow degradation of the alloy implant due to the inflammatory situation and a higher degradation of the alloy implants under the synergistic effects of both compounds added to a biological solution.

## 2. Materials and Methods

An electrochemical workstation, a VoltaLab PGZ 301 controlled by VoltaMaster4 software (5.10, Radiometer Analytical, Lyon, France), was used for in vitro electrochemical measurements. An electrochemical cell consisting of three electrodes (Ag/AgCl as reference electrode, Pt-Rh grid as the counter electrode, and a working electrode made from Ti6Al4V grade 5 with a constant volume of electrolyte (250 mL)) was used in each electrochemical measurement. The tests were performed four times to observe the reproducibility of the obtained data. The samples were not removed from the solution for 168 h. The alloy samples were prepared with a copper wire electrical contact and insulated with epoxy resin in order to obtain a very well-defined active surface area of 5.25 ± 0.1 cm^2^ for each tested sample. The roughness of the alloy surface of Ra = 1.15 ± 0.03 µm is maintained as it was received from customer. Before immersion, each sample was degreased in acetone and ethylic alcohol and then dried [17]. The chemical composition of grade 5 Ti6Al4V alloy is N—0.003%, Al—5.86%, C—0.008%, V—3.73%, H—0.002%, Fe—0.068%, O—0.084%, Ti—89.976%; max. N—0.003%, Al—6.01%, C—0.008%, V—3.83%, H—0.002%, Fe—0.083%, O—0.088%, and Ti—90.245% [17]. The base electrolyte used for in vitro corrosion investigation is a biological Hanks’ solution [29]. In order to simulate the inflammatory conditions and metabolic products from the oral cavity or produced by the muscles during physical effort Hanks’ solution was modified by adding hydrogen peroxide, H_2_O_2_ (30% p.a.) and lactic acid, LA, (85% p.a., Sigma Aldrich, Saint Louis, MO, USA). With the aid of a pH meter, Sension+, the important parameters of each solution were measured as: the pH, the conductivity, and the salinity, as shown in Table 1. This experiment was carried out at a temperature of 37 °C ± 0.5 in order to simulate the real condition of the human body.

**Figure 1:**
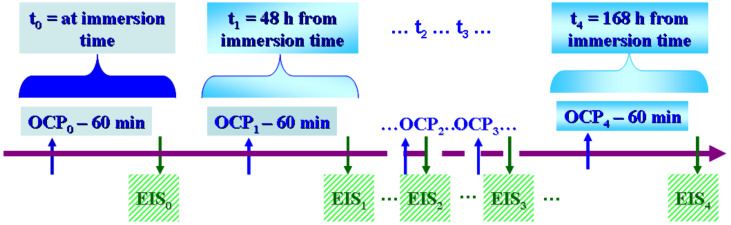
Schematic drown of electrochemical measurements protocol for in vitro corrosion investigations of grade 5 Ti6Al4V alloy.

The electrochemical investigations applied for corrosion evaluation measurements are presented schematically in Figure 1.

At the time of immersion of the sample into a specific solution, noted as t_0_, an open circuit potential was performed for 60 min with a measurement period of 0.2 sec expressed as OCP_0_, as shown in Figure 1, followed by an electrochemical impedance spectroscopy measurement (at free potential from 100 kHz to 1 mHz with a sinusoidal amplitude of 10 mV and 20 frequency per decade) noted as EIS_0_. The sequence of open circuit potential and electrochemical impedance spectroscopy measurements was performed at periods of time at multiples of 12 h as follows:

At t_1_ = 48 h, OCP_1_ and EIS_1_ were measured;
At t_2_ = 120 h, OCP_2_ and EIS_2_ were measured;
At t_3_ = 144 h, OCP_3_ and EIS_3_ were measured;
At t_4_ = 168 h, OCP_4_ and EIS_4_ were measured.

Figure 1 schematically presents the measurements performed at t_0_ = immersion time, t_1_ = 48 h, and t_4_ = 168 h. In the Results Section we present Figures of the cumulated time effect on open circuit potential and the electrochemical impedance spectroscopy as a Nyquist representation for every solution at every measured time. The Bode representations of electrochemical impedance spectroscopy are shown only at immersion time, t_0_, and after 168 h, t_4_. For the other EIS measurements, we present only the specific resistance values obtained in order to complete the evolution of EIS during the total immersion period. Separately from the used sample for the electrochemical measurements, different samples were immersed only into the specified solutions in order to observe them using SEM-EDX in order to see the effect of biological solutions and the added components on the surface of the grade 5 titanium alloy. The immersion time was 168 h. After this period, the samples were cleaned with distilled water, dried, and observed using SEM-EDX.

A FEI QUANTA 200 scanning electron microscope (SEM, FEI Company, Hillsboro, OR, USA) was used to determine and compare the morphological variations of the studied samples of grade 5 Ti6Al4V alloy, tested in Hanks’ solution, Hanks’ solution + 10 g/L lactic acid, Hanks’ solution + 5 g/L H_2_O_2_, and the synergistic effect of hydrogen peroxide and lactic acid (Hanks’ solution + 10 g/L lactic acid + 5 g/L H_2_O_2_)). In addition, the samples were analyzed in terms of their elemental composition by energy-dispersive X-ray spectroscopy (EDX) with the help of EDAX Genesis data acquisition software.

X-ray diffraction spectra (XRD) were recorded using the Dron 3 equipment (Bourevestnik Inc., St. Petersburg, Rusia) and a Cobalt anode (Co, λ_Ka_ = 1.790300 Ǻ, Bourevestnik Inc., St. Petersburg, Rusia) with setting of: voltage (U) of 30 kV and an intensity (I) of 20 mA, with a step of 0.05°/s and a range between 15–90°. The obtained spectra were correlated using standardized software data Match! 3 (3.12, Crystal Impact, Bonn, Germany) for phase identification (http://www.crystalimpact.com/match, accessed date 25 September 2021). The results were correlated with the Crystallography Open Database (COD, 2021, Crystal Impact, Bonn, Germany), identifiable with a 9-digit code.

## 3. Results and Discussions

### 3.1. Open Circuit Potential Evolution during Immersion Time (OCP)

**Figure 2:**
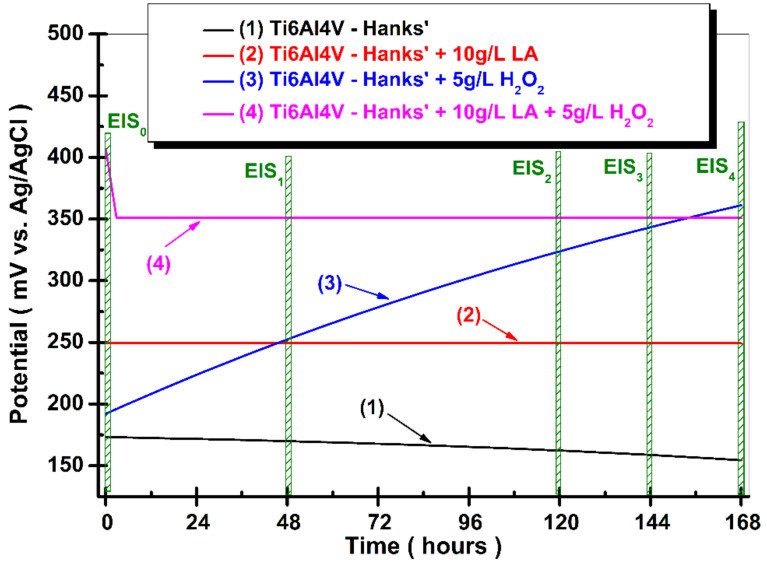
Evolution of open circuit potential for Ti6Al4V alloy during 168 h from immersion time in: (1) Hanks’ solution; (2) Hanks’ solution + 10 g/L lactic Acid (LA); (3) Hanks’ solution + 5 g/L H_2_O_2_, and (4) Hanks’ solution + 10 g/L LA + 5 g/L H_2_O_2_.

Figure 2 shows the OCP evolution (potential vs. time) of Ti6Al4V alloy during 168 h from immersion time in four studied solutions.

From Figure 2 it can be observed that the potential values of the samples are different because of oxide film formation on the surface of the Ti6Al4V implant biomaterial. A slow and small shift of the potential to more negative values is observed for the Ti6Al4V alloy tested in Hanks’ solution, which begins with 173.20 mV vs. Ag/AgCl at the immersion time before reaching a value of 154.46 mV vs. Ag/AgCl at the end of the measurement, which is a less noble curve (1).

This trend could show a slow dissolution (instability) of the oxide film formed on the surface of the tested biomaterial. In the case of Ti6Al4V immersed in Hanks’ solution with added lactic acid (Figure 2, curve (2)) it can be observed that the value of the open circuit potential reaches the equilibrium state from the immersion time and the value of the open circuit potential is constantly maintained until the end of the measurement at a value of 249.31 mV vs. Ag/AgCl. This behavior is justified by the fact that lactic acid is absorbed onto the surface of the alloy biomaterial that is acting as a protective barrier. This behavior was also observed by Qing Qu et al. [27] when studying the corrosion behavior of titanium for 14 days after immersion in artificial saliva with the addition of lactic acid. The adsorption of lactic acid on the surface of the titanium alloy is further proven by X-ray diffraction patterns by the specific peak. As can be seen in Figure 2 curve (3), a higher shift and increasing trend of the potential value for titanium alloy immersed in Hanks’ solution with an added H_2_O_2_ inflammatory compound to a more positive value is observed. The starting value, at immersion time, of the open circuit potential is E = 192.11 mV vs. Ag/AgCl before reaching after 168 h the value of E = 361.17 mV vs. Ag/AgCl, which is more positive (more noble).

This behavior was observed by other authors and could be explained by the formation of a layer of a thin film of TiO_2_, because it is known that hydrogen peroxide is a strong oxidizing compound [14,25,32,33,34]. The addition of hydrogen peroxide promotes the growth of TiO_2_ on the surface of an alloy biomaterial, but it can also have the effect of dissolving the oxide formed on the surface of the material following exposure to oxygen, as expressed by the possible reactions previously considered by some authors [35,36]:

(1) $$Ti+H_{2}O_{2}\;\to \;TiO_{2}+H_{2}\;$$

(2) $$2H_{2}O_{2}\to 2H_{2}O+O_{2}$$

(3) $$TiO_{2}+2H_{2}O\;\to \;Ti{\left(OH\right)}_{4}\;$$

(4) $$TiO_{2}+nH_{2}O\;\to \;TiO_{2}nH_{2}O\;$$

(5) $$\left(\mathrm{Ti}-\mathrm{OH}\right)+{\;H}_{2}O\;\to {\left[\mathrm{Ti}-O\right]}^{-}+H_{3}O^{+}$$

(6) $$TiO_{2}nH_{2}O+OH^{-}\;\to HTiO_{3}^{-}nH_{2}O\;$$

Titanium from an alloy could react with hydrogen peroxide to form a titanium oxide film (Equation (1)). Further, some TiO_2_ could be hydrolyzed (Equations (2) and (3)). A hydration reaction (Equation (4)) is occurring simultaneously with the formation of Ti-OH functional groups (Equation (5)). Hydrated and hydrolyzed titanium oxide (TiO_2_) can cause the formation of negatively charged surfaces (Equations (5) and (6)), which could be involved in decreasing the open circuit potential to a more negative value at immersion time followed by continuously shifting to a more positive value, as explained by Equation (1). This behavior is also sustained by the EIS results.

For Ti6Al4V tested in Hanks’ solution with an added mixture of LA and H_2_O_2_, the open circuit potential value shows a decreasing trend in the first 3 h from 406.78 mV vs. Ag/AgCl to 350.90 mV vs. Ag/AgCl. After this period, the open circuit potential value is maintained at a constant value until the end of the 168 h at E = 350.90 mV. The lactic acid adsorbed on the alloy surface partially inhibits the reactions between the alloy surface and hydrogen peroxide.

### 3.2. Electrochemical Impedance Spectroscopy

Electrochemical impedance spectroscopy used in this study is an electrochemical method where an alternating current is used to characterize the processes taking place at the electrode–electrolyte interface.

For the analysis of electrochemical systems, the electrochemical impedance spectroscopy method can provide a wide range of information about the kinetics of the processes that take place at the electrode–electrolyte interface; thus, this method is used in the study of corrosion processes [36], to characterize semiconductors [37], in the study of batteries [38], and in the study of the kinetics of electrochemical deposition [39] or electrocatalysis [40] processes.

In the reactivity and corrosion studies of metallic surfaces, electrochemical impedance spectroscopy diagrams (EIS) provide complete information about the kinetics of complicated processes (reactions) that take place at the electrode–electrolyte interface (the corrosive environment in which the studied material is immersed) [41,42].

EIS diagrams can be presented both in the complex plane (Nyquist)—when the real part is represented on the abscissa and the imaginary part is represented on the ordinate—and in the Bode plane—when the logarithmic frequency is represented on the abscissa and the logarithmic impedance modulus or phase angle are represented on the ordinate.

Complex representation is often used in the literature because it allows for the easy identification of the equivalent circuit elements that are used to fit the recorded experimental data and determine the polarization or the specific resistance.

**Figure 3:**
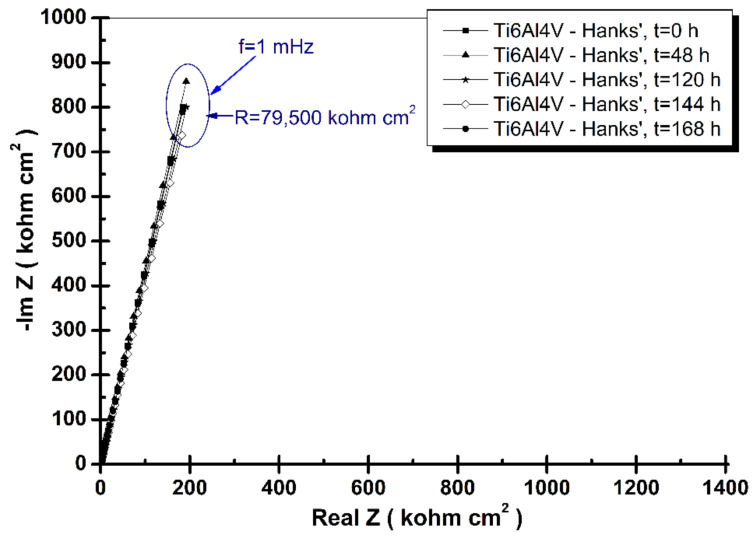
Nyquist spectra of electrochemical impedance spectroscopy results of grade 5 titanium alloy immersed in Hanks’ biological solution obtained during periodic measurements at: t_0_, t_1_, t_2_, and t_4_. The line represents the fitted results; the experimental points are the symbols.

**Figure 4:**
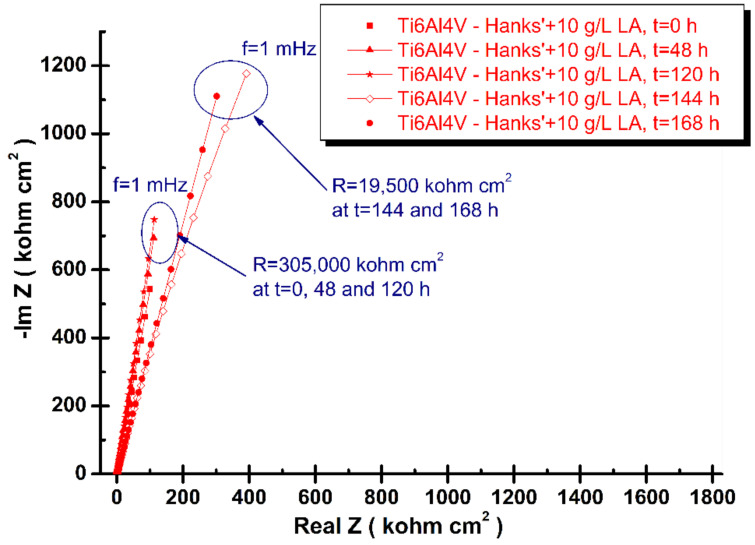
Nyquist spectra of electrochemical impedance spectroscopy results of grade 5 titanium alloy immersed in Hanks’ solution + 10 g/L lactic acid obtained during periodic measurements at: t_0_, t_1_, t_2_, t_3_, and t_4_. The line represents the fitted results; the experimental points are the symbols.

**Figure 5:**
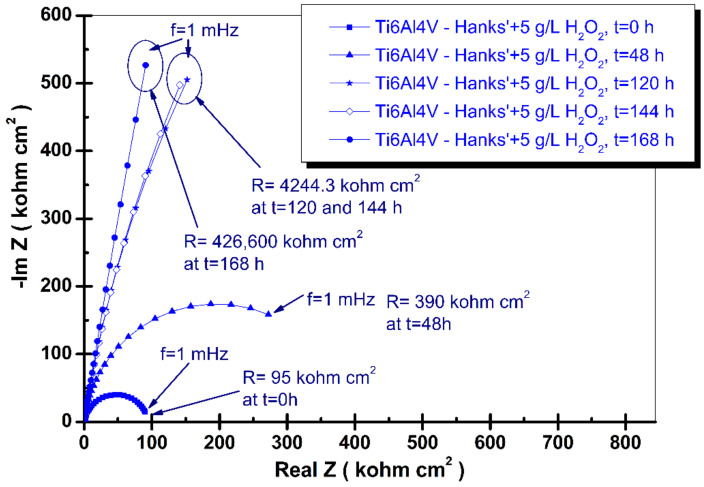
Nyquist spectra of electrochemical impedance spectroscopy results of grade 5 titanium alloy immersed in Hanks’ solution + 5 g/L hydrogen peroxide (H_2_O_2_) obtained during periodic measurements at: t_0_, t_1_, t_2_, t_3_, and t_4_. The line represents the fitted results; the experimental points are the symbols.

**Figure 6:**
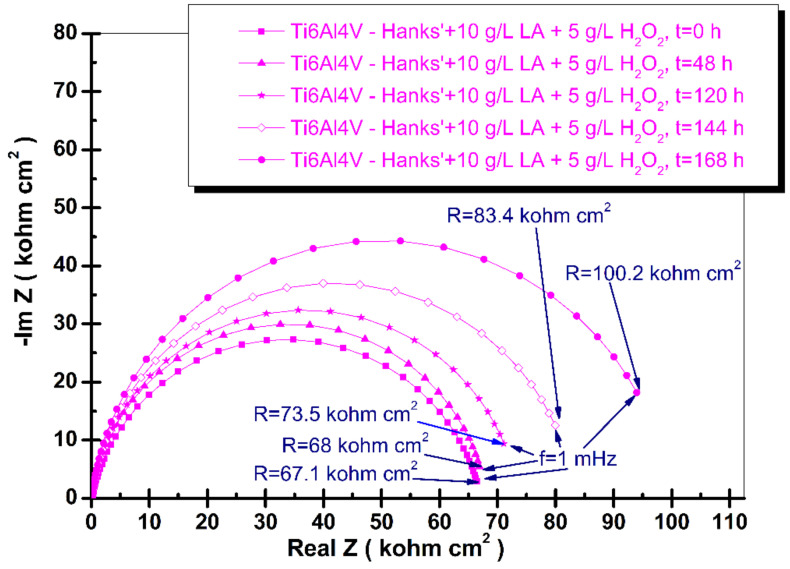
Nyquist spectra of electrochemical impedance spectroscopy results of grade 5 titanium alloy immersed in Hanks’ solution 10 g/L lactic acid+ 5 g/L hydrogen peroxide (H_2_O_2_) obtained during periodic measurements at: t_0_, t_1_, t_2_, t_3_, and t_4_. The line represents the fitted results; the experimental points are the symbols.

Due to the porosity and/or inhomogeneity of the surfaces subjected to corrosion tests [43,44] only simple elements (e.g., resistors, capacitors, inductors, etc.) could not be used to fit the EIS diagrams in the equivalent circuits, so it is necessary to use a constant phase element (*CPE*), which allows a representation of the frequency dispersion, the value of which is determined according to the relation [45].

(7) $$Z_{CPE}=\frac{1}{Q{\left(j\omega \right)}^{\alpha }}$$

where: *ω* is the angular frequency (*ω* = 2πf, f is the frequency in Hz), *Q* is a real constant frequency independent (F cm^−2^), and α is dependent on the rotation angle of a pure capacitor in a complex plane. The complex Nyquist diagrams of electrochemical impedance spectroscopy results of grade 5 titanium alloy immersed in the tested four biological solutions recorded from immersion time, t_0_, to the final time, t_4_ (168 h), are shown in Figure 3, Figure 4, Figure 5 and Figure 6.

From Figure 3 it can be observed that grade 5 titanium alloy is relatively stable in Hanks’ biological solution, having practically the same specific resistance value measured for entire tested period, R = 71,500–79,500 kohm·cm^2^. The Nyquist EIS diagrams are practically superposed for each measured period. Constant values of polarization resistance in Hanks’ solution obtained by EIS method were also observed by other authors who studied the effect of H_2_O_2_ on the electrochemical behavior of Ti-13Nb-13Zr alloy for 60 days [46].

As can be seen in Figure 4, the lactic acid addition affects the electrochemical impedance spectroscopy diagrams recorded during the immersion period of 168 h. At the first three periods of measurements, t = 0, 48, and 120 h) the specific resistance is constantly showing essentially the same values. A slow decreasing of specific resistance is observed after 144 and 168 h from immersion time.

This decrease could be explained by the possible reaction between titanium and lactic acid [27] that forms a lactate chelating compound that has an accelerating effect on the dissolution of oxide film. This has been found by other authors studying corrosion behavior of titanium in artificial saliva with lactic acid for 14 days [27].

(8) $$\left[\mathrm{Ti}{\left(\mathrm{OH}\right)}_{3}\right]\cdot \mathrm{Cl}+L^{-}\to \left[\mathrm{Ti}{\left(\mathrm{OH}\right)}_{3}\right]\;\cdot L+{\mathrm{Cl}}^{-}$$

where: L is lactate anion (L^−^) and Cl^−^ is chloride anion.

The addition to Hanks’ solution with hydrogen peroxide, the inflammatory compound, affects the electrochemical impedance spectroscopy results recorded, as is shown in Figure 5.

The first measured specific resistance (t = immersion time) is smaller than the previous measured in Hanks’ solution and Hanks’ solution with added lactic acid at the same immersion time. The specific resistance value is 95 kohm·cm^2^ but has a tendency to increase during the immersion period, confirming the possible reactions presented as Equations (1)–(6). Thus, the value of the specific resistance increases to 390 kohm·cm^2^ at an immersion time of 48 h, to 4244.3 kohm·cm^2^ at 120 and 148 h, before reaching the highest value after 168 h of 426,600 kohm·cm^2^.

This behavior suggests that the alloy surface immersed in Hanks’ solution under inflammatory conditions is oxidized but the formed oxide is further dissolved and is thus unstable and does not protect the bulk alloy material. Furthermore, during a longer immersion period the alloy surface could be protected by the newly formed oxide (Equations (1)–(6)).

This behavior was also observed by other authors who studied the effect of H_2_O_2_ on the electrochemical behavior of a Ti-13Nb-13Zr alloy in Hanks’ solution for 60 days [46].

They showed that initially the effect of the corrosive attack of H_2_O_2_ on the oxide film was mainly noticed through a decrease of the barrier layer’s impedance. For longer immersion periods, the H_2_O_2_ concentration diminishes due to consumption, and in the H_2_O_2_ depleted solution the oxide film recovers its protective characteristics that could occur by incorporation of precipitates or ions into the film, thus resulting in the increase in impedance results [46].

When both compounds are added to Hanks’ biological solution, their synergistic effect on the degradation of the titanium alloy implant is drastically higher. Thus, the first specific resistance measured at immersion time is much lower, having a value of 67.1 kohm·cm^2^, as can be observed in Figure 6. The specific resistance increases very slowly during the immersion time with a value of 100.2 kohm·cm^2^, in the same order of magnitude at each measured period. The low specific resistance of the alloy implant resulted in an inflammatory situation combined with the presence of lactic acid, which suggests that the Ti6Al4V implant replacement become unstable and was affected by the degradation process due to the corrosion of this complex environment. The titanium oxide film is reformed much slower on the titanium alloy surface (Equation (1)) due to lactic acid being adsorbed.

**Figure 7:**
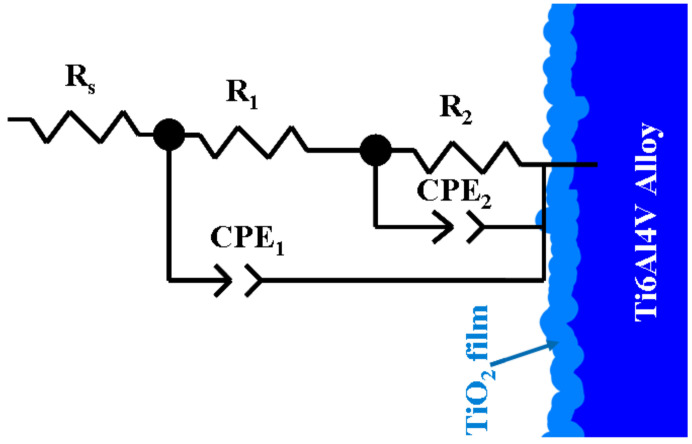
Electrical equivalent circuit proposed to fit the interface of Ti6Al4V implant alloy and Hanks’ biological solution as well as Hanks’ solution with added compounds.

In order to obtain the specific resistance values and other specific data, the electrochemical impedance spectroscopy results were simulated and fitted with Zview software. The better electrical equivalent circuit proposed for grade 5 titanium alloy surface immersed in all four tested solutions is presented in Figure 7. The chi-squared result obtained after fitting and simulation is 10^−3^, confirming that the experimental results are well fitted.

Instead of a double layer pure capacitance, C_dl_, a constant phase element (CPE) is used. Thus, the meanings of circuit elements are as follows:

- R_s_ is the solution resistance;
- R_1_ is the resistance of thin passive oxide layer formed on titanium alloy, which is in direct contact with the tested biological solution;
- CPE_1_ is the constant phase element corresponding to this thin passive oxide film;
- R_2_ is the higher resistance of bulk alloy implant, which is in contact with the tested solution through the oxide film;
- CPE_2_ is the constant phase element corresponding to the interface of bulk titanium alloy and the biological solution through the passive oxide film. The fitted parameter values for each system of titanium alloy and the tested biological solutions are shown in Table 2. The similar equivalent circuit was found by other authors [47] when studying the corrosion resistance of Ti6Al7Nb and Ti6Al4V ELI in an SBF solution.

**Figure 8:**
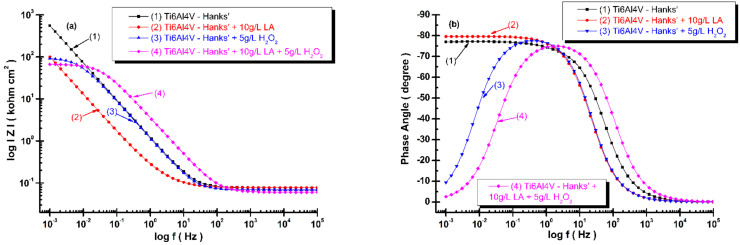
Bode spectra of electrochemical impedance spectroscopy results of grade 5 titanium alloy immersed in all four biological solutions obtained during immersion time measurements, t_0_: (**a**) log $\left|Z\right|$ versus log (frequency); (**b**) phase angle versus log (frequency). The line represents the fitted results; the experimental points are the symbols.

**Figure 9:**
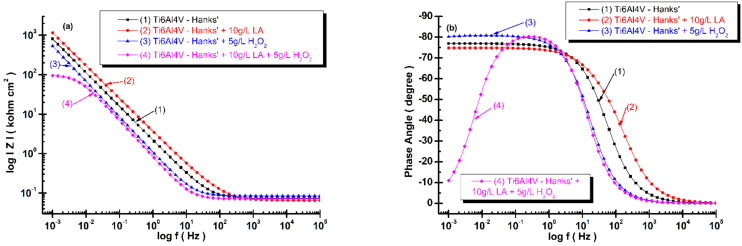
Bode spectra of electrochemical impedance spectroscopy results of grade 5 titanium alloy immersed in all four biological solutions obtained during immersion period after 168 h, t_4_: (**a**) log $\left|Z\right|$ versus log (frequency); (**b**) phase angle versus log (frequency). The line represents the fitted results; the experimental points are the symbols.

The electrical equivalent circuit and the fitted parameters of the Bode spectra of electrochemical impedance spectroscopy results (as symbols) obtained for each system (alloy and solution), recorded at t_0_ and t_4_, and the fitted diagrams (line) are shown in Figure 8a,b and Figure 9a,b.

In the Bode plot, the logarithm of the total impedance log *Z* (or impedance modulus (magnitude) $\left|Z\right|$), is plotted versus the logarithm of the frequency log f. Additionally, the phase shift, θ, is plotted versus the log f. The advantage of this is that the frequency dependence is clearly visible in the Bode plot and no information is lost.

The impedance magnitude and impedance phase angle are given by following equations:

(9) $$\left|Z\right|=\sqrt{{\left(Z_{real}\right)}^{2}+{\left(Z_{im}\right)}^{2}}$$

(10) $$\theta =\tan^{-1}\left(\frac{Z_{im}}{Z_{real}}\right)$$

From Figure 8a and Figure 9a, the slope of the transition region between the two asymptotic limits reveals the power of the frequency dependence of the imaginary part of the impedance results. It can be observed that the slopes of each diagram are very similar and close to 0.9 values.

Figure 8a shows the impedance magnitude of the titanium alloy at the time of immersion (t_0_) in Hanks’ solution is higher as compared with the impedance magnitude resulting from all the modified Hanks’ solutions; the lowest one is the impedance magnitude for titanium alloy immersed in Hanks’ solution modified with mixed lactic acid and hydrogen peroxide. The phase angle values, Figure 8b, are close to −80 degrees for titanium alloy immersed in Hanks’ solution and Hanks’ solution modified with lactic acid on a larger frequency domain, confirming a slow reactivity and a good corrosion resistance of titanium alloy in these solutions. At time of the immersion, t_0_, for the other two solutions—Hanks’ solution with H_2_O_2_ and Hanks’ solution modified with the mixture of the two compounds—the phase angle is located at the lowest values, which are below −10 degrees. These behaviors confirm a higher reactivity of titanium alloy and a degradation effect by corrosion process occurring on the titanium alloy’s surface.

Analyzing the Bode plots after 168 h, Figure 9, it can be observed that the impedance magnitude of the titanium alloy immersed in Hanks’ solution remains at about the same value and the impedance magnitude of titanium alloy immersed in Hanks’ solution with added lactic acid and Hanks’ solution with added hydrogen peroxide reach about the same order of magnitude values, as seen in Figure 9a. The surface of the titanium alloy immersed in Hanks’ solution with hydrogen peroxide shows self-healing behaviors after 168 h. Its impedance magnitude increases by about one order of magnitude compared with the value recorded at time of immersion, t_0_, as shown in Figure 9a. The impedance magnitude of titanium alloy immersed in Hanks’ solution with added mixed compounds is the only one at a lower value after 168 h, being about at same order of magnitude as it was recorded at time of immersion, t_0_, confirming that Equation (1) to reform the titanium oxide film is inhibited by lactic acid adsorbed on the titanium alloy’s surface.

The phase angle after 168 h shows the same behavior of titanium alloy immersed in three solutions, respectively, Hanks’ solution, Hanks’ solution with added lactic acid, and Hanks’ solution with added hydrogen peroxide, which is close to −80 degrees on a large frequency domain (Figure 9b). The phase angle of the titanium alloy immersed in Hanks’ solution with mixed lactic acid and hydrogen peroxide shows the same low value, −10 degrees, which is close to the value recorded at time of immersion, t_0_, thus confirming the inhibition of reforming titanium oxide film.

### 3.3. SEM–EDX Analysis before and after Corrosion

**Figure 10:**
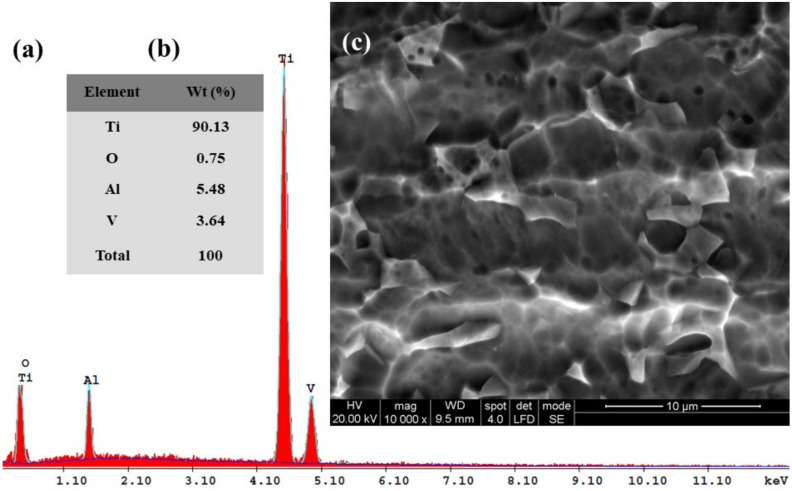
SEM surface micrographs and EDX analysis of the Ti6Al4V alloy implant before corrosion tests: (**a**) EDX spectra; (**b**) the EDX elemental analysis; (**c**) SEM surface morphology.

**Figure 11:**
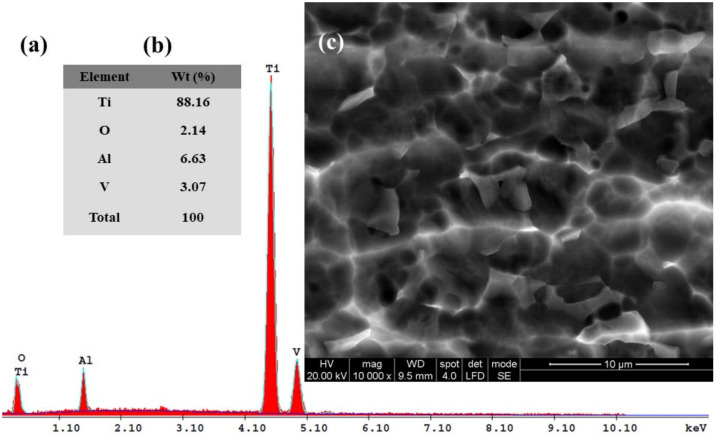
SEM surface micrographs and EDX analysis of Ti6Al4V alloy after 168 h from immersion time in biological Hanks’ solution: (**a**) EDX spectra; (**b**) the EDX elemental analysis; (**c**) SEM surface morphology.

**Figure 12:**
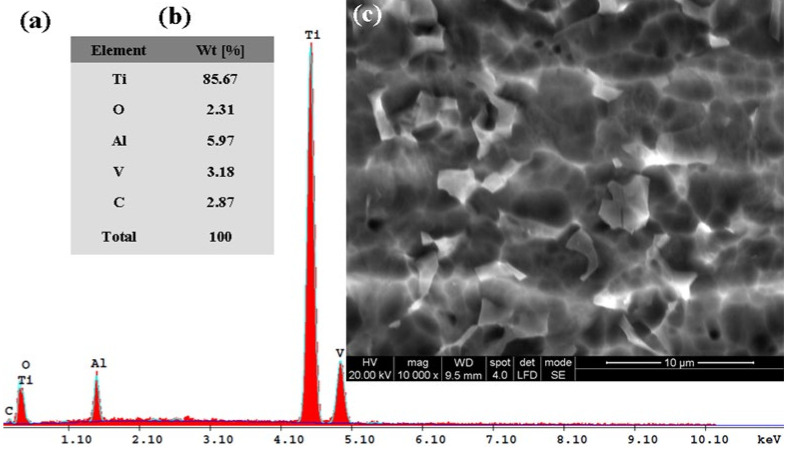
SEM surface micrographs and EDX analysis of Ti6Al4V alloy after 168 h from immersion time in biological Hanks’ solution + 10 g/L lactic acid (LA): (**a**) EDX spectra; (**b**) the EDX elemental analysis; (**c**) SEM surface morphology.

**Figure 13:**
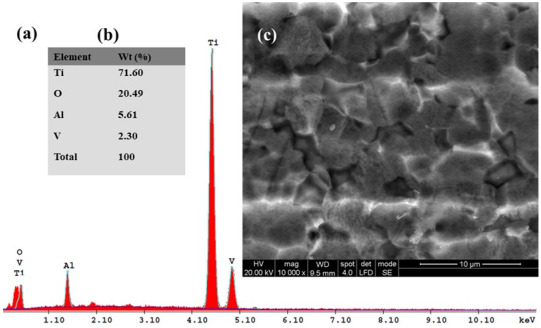
SEM surface micrographs and EDX analysis of Ti6Al4V alloy after 168 h from immersion time in biological Hanks’ solution + 5 g/L H_2_O_2_: (**a**) EDX spectra; (**b**) the EDX elemental analysis; (**c**) SEM surface morphology.

**Figure 14:**
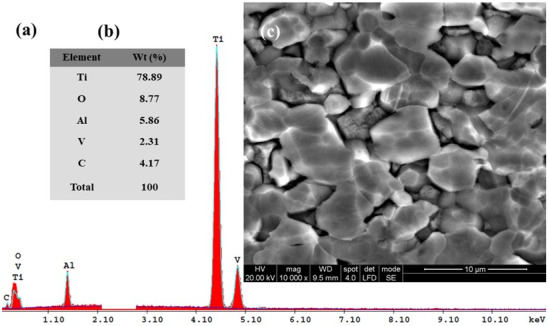
SEM surface micrographs and EDX analysis of Ti6Al4V alloy after 168 h from immersion time in biological Hanks’ solution + 10 g/L LA + 5 g/L H_2_O_2_: (**a**) EDX spectra; (**b**) the EDX elemental analysis; (**c**) SEM surface morphology.

Subsequently, for the immersed samples in all four tested solutions and for Ti6Al4V metallic biomaterials before the corrosion experiments, the SEM-EDX analyses with elemental composition were performed. The results are shown in Figure 10, Figure 11, Figure 12, Figure 13 and Figure 14.

The EDX analysis of the Ti6Al4V implant alloy before corrosion (Figure 10a,b) indicates the presence of the main elements from the studied biomaterials, respectively: Ti, Al, and V, with their relative atomic percentage values of (wt.%), respectively, Ti—90.13%, Al—5.48%, and V—3.64%. The oxygen from the thin titanium oxide native film is 0.75%. This small amount of oxygen indicates that the very thin passive oxide film on the titanium alloy’s surface formed as a native oxide before the corrosion tests.

Figure 11, Figure 12, Figure 13 and Figure 14 present the SEM surface micrographs of the Ti6Al4V alloy performed 168 h after the initial immersion time studied in the four biological solutions. The EDX elemental analyses corresponding to the tested samples.

From a morphological point of view, the alloy samples immersed in Hanks’ solution and Hanks’ solution with added lactic acid did not show any corrosion degradation (Figure 11c and Figure 12c).

From Figure 13c, a change in the morphology of the studied surfaces due to the presence of hydrogen peroxide in the Hanks’ solution (5 g/L H_2_O_2_) is observed. The morphology shows an irregular polyhedral surface that is not well defined and some degradation from corrosion as well as a surface similar to one that is covered with a thin oxide film. This fact is due to the strong oxidizing effect of hydrogen peroxide, which increases the appearance of TiO_2_ film on the sample surface combined with a corrosion attack, explained by the reactions that occur on the titanium alloy’s surface, as described by Equations (1)–(6). The surface morphology confirms the EIS results obtained for each biological solution.

In the case of the titanium alloy immersed in Hanks’ solution with added lactic acid mixed with hydrogen peroxide, Figure 14c, there is an obvious tendency towards degradation of the alloy’s surface by the formation of intergranular voids through dissolution of some material. The SEM micrograph confirms the results obtained by electrochemical impedance spectroscopy measurements from Figure 6 and Figure 9a,b with smaller specific resistance for the Ti6Al4V alloy implant immersed in mixed Hanks’ solution with added lactic acid and hydrogen peroxide, as compared with the results obtained for the same alloy immersed in the other tested solutions.

From the EDX analysis of the Ti6Al4V alloy immersed in Hanks’ solution, Figure 11a,b, it can be observed that in comparison with the EDX analysis of Ti6Al4V before the corrosion process, the atomic relative mass of Ti decreases to 88.16%. Additionally, an increase of the oxygen element by 2.14% is observed, which indicates that an oxidation effect on the titanium alloy occurs when the alloy’s surface is immersed in Hanks’ biological solution.

Similar EDX spectra and chemical analyses are seen in Figure 12a,b when the titanium alloy is immersed in Hanks’ solution with added lactic acid. The lactic acid addition did not affect the oxidation state of the Ti6Al4V alloy; the oxygen content was about the same at 2.31%. The carbon element appears in the EDX spectra and chemical analysis, Figure 12a,b, at a content value of 2.87%, thus confirming the adsorption of lactic acid (organic molecule) on the Ti6Al4V alloy surface when it is immersed in Hanks’ solution with added lactic acid.

Figure 13a,b shows the EDX spectra and chemical analysis of the titanium alloy’s surface immersed in Hanks’ solution with added hydrogen peroxide, which confirms the strong oxidizing effect of hydrogen peroxide that favors the formation of titanium dioxide (TiO_2_). This confirms that Equation (1) is occurring on the alloy surface. This fact is demonstrated by the decreasing trend of the mass percentages of the Ti element to 71.60% and the increase of the mass percentage of the oxygen element to 20.49%, as compared to the titanium alloy surface immersed in Hanks’ solution. The results confirm the reactions expressed by Equations (1)–(6) occurring on the titanium alloy surface as well as the EIS results obtained.

From the EDX spectra and chemical analysis of Ti6Al4V alloy surface immersed in Hanks’ solution with added mixture of lactic acid and an inflammatory compound, a decreasing trend of mass percentage of Ti element to 78.89%, an increasing trend of oxygen element to 8.77% and the apparition of carbon element at 4.17% can be seen. At the same time, the oxygen element shows a decreasing trend of the oxygen mass percentage to 8.77% when compared with the alloy surface immersed in Hanks’ solution with added hydrogen peroxide. This is due to the lactic acid’s adsorption on the alloy surface, which probably protects from further oxidation but also provokes the corrosion process to occur on the alloy implant’s surface.

For the carbon element recorded in this biological solution at a value of 4.17% as identified by EDX analysis, it is revealed that there is an increasing trend of the mass percentage as compared with Hanks’ solution with only added lactic acid. It is likely that the added hydrogen peroxide compound forms an oxide film and that it also increases the adsorption of the lactic acid molecules. Both compounds combine their effect in a synergistic degradation effect by creating a corrosive attack on the alloy implant’s surface. At the same time, from EDX analyses shown in Figure 10a,b, Figure 11a,b, Figure 12a,b, Figure 13a,b and Figure 14a,b all show that the identified elements have similar positions on the EDX spectra.

### 3.4. X-ray Diffraction Patterns of the Ti6Al4V before and after Immersion

**Figure 15:**
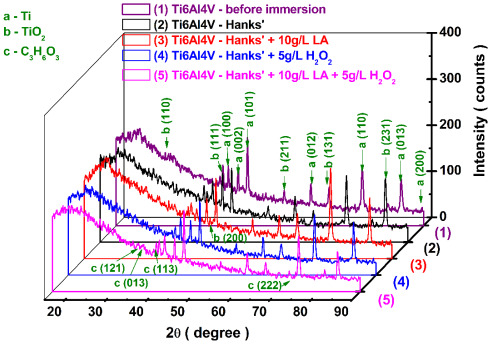
XRD patterns of Ti6Al4V biomaterial implant alloy: (1) before immersion and corrosion experiment and after 168 h from immersion time: (2) Hanks’ solution; (3) Hanks’ solution + 10 g/L lactic acid (LA); (4) Hanks’ solution + 5 g/L H_2_O_2_; and (5) Hanks’ solution + 10 g/L LA + 5 g/L H_2_O_2_.

The X-ray diffraction patterns (XRD) of the Ti6Al4V biomaterial implant alloy before the corrosion experiments and after immersion in the four biological solutions are presented in Figure 15, curve (1).

From the analysis of the XRD patterns (Figure 15, curve (1)) of the Ti6Al4V alloy before the corrosion tests with the help of the Match! 3 software, the following crystalline phases were identified: (a) titanium (Ti) with crystallographic plans ((100), (002), (101), (012), (110), (013), and (200)) corresponding to angles 2θ (42.33°, 44.68°, 47.17°, 62.61°, 74.90°, 84.49°, and 89.20°). (b) Titanium dioxide (TiO_2_) with crystallographic plans ((111), (002), (211), (131), (231), (023), and (200)) corresponding to angles 2θ (40.24°, 41.03°, 55.93°, 67.05°, 81.30°, 78.82°, and 45.87°).

Additionally, in the background the following patterns were identified: aluminum (Al) with crystallographic plans ((200) and (202)), corresponding to angles 2θ (49.00° and 76.86°). Another phase identified in the background was vanadium (V) with crystallographic plans (200), corresponding to angles 2θ (74.12°).

Figure 15 also shows the XRD patterns of the Ti6Al4V alloy obtained on the surfaces after 168 h from immersion period in: (2) Hanks’ solution; (3) Hanks’ solution + 10 g/L lactic acid (LA); (4) Hanks’ solution + 5 g/L H_2_O_2_; and (5) Hanks’ solution + 10 g/L LA + 5 g/L H_2_O_2_.

By analyzing, the XRD patterns obtained on the alloys’ surfaces after 168 of immersion and corrosion experiments done in the tested solutions, we can see the preservation of the diffraction angles and the phases identified for Ti, Al, V, and TiO_2_, as in the case of the titanium alloy before corrosion.

On the studied surfaces we identified titanium (Ti) with crystallographic plans ((100), (002), (101), (012), (110), (013), and (200)) corresponding to angles 2θ (42.33°, 44.68°, 47.17°, 62.61°, 74.90°, 84.49°, and 89.20°). This detected phase is recorded in the database of the program we used, Crystallography Open Database (COD) 96-901-6191, and belongs to the hexagonal crystallization system, space group P63/mmc. Aluminum (Al) is recorded in the program’s database COD 96-431-3211, which belongs to the cubic crystallization system, space group F m -3 m with crystallographic plans ((200) and (202)), corresponding to angles 2θ (49.00° and 76.86°).

Another phase identified was vanadium (V) with crystallographic plans (200), corresponding to angles 2θ (74.12°). This detected phase is recorded in the program’s database Crystallography Open Database (COD) 96-410-5684 and it belongs to the cubic crystallization system, space group I m -3 m.

Titanium dioxide (TiO_2_) recorded in the program’s database COD 96-153-0027, which belongs to the orthorhombic crystallization system, space group (P b c n) with crystallographic plans ((110), (111), (002), (211), (202), (221), (131), (231), (023), and (200)) corresponding to angles 2θ (28.08°, 40.24°, 41.03°, 55.93°, 63.92°, 64.75°, 67.05°, 81.30°, 78.82°, and 45.87°).

In Figure 15, curve (4) and curve (5), the last phase identified is lactic acid (C_3_H_6_O_3_), recorded in the program’s database COD 96-500-0200 with crystallographic plans ((111), (102), (121), (013), (113), (213), (232), (222), and (111)) corresponding to angles 2θ (22.19°, 23.50°, 30.04°, 33.18°, 35.67°, 40.37°, 50.57°, 60.12°, and 70.06°), which belongs to the orthorhombic crystallization system, space group (P 21 21 21).

The lactic acid phase identified by XRD on the titanium alloy surface after immersion in Hanks’ solution + LA and Hanks’ solution + LA + H_2_O_2_ proves once more that the lactic acid is adsorbed on the titanium alloy’s surface and affects the reactivity as well as the corrosion resistance; therefore, this confirms electrochemical results (OCP and EIS).

The XRD patterns provide information about the crystal structure of the samples observed after immersion in the tested solutions, the formation of titanium dioxide on the surface of the titanium alloy samples, and the absorption of lactic acid on the alloy’s surface.

As can be seen from Figure 15, on the Ti6Al4V samples analyzed before and after corrosion in the four solutions, titanium dioxide (TiO_2_) appeared with significant diffraction peaks on the XRD diagram. This proves that the surface of the titanium alloy oxidizes and predominantly forms a passive TiO_2_ film.

From the XRD diagrams, it is observed that TiO_2_ electro-crystallizes preferentially according to the planes ((211), (111), and (131)), which have the highest intensity. The peak corresponding to the crystallization plane (211) of TiO_2_ increases in intensity with the immersion of the titanium alloy in the Hanks’ solution, and with the addition of lactic acid in the Hanks’ solution, compared to the non-immersed alloy, but a decrease in the peak is observed with the addition of hydrogen peroxide and the two compounds in combination (hydrogen peroxide + lactic acid).

The second crystallization plan of TiO_2_, (111), decreases slightly in intensity with the addition of both lactic acid and hydrogen peroxide, as well as the two mixed compounds in Hanks’ solution, compared to Ti6Al4V immersed in Hanks’ solution.

However, there is a slight increase in the peak according to the crystallization plan (111) with the presence of hydrogen peroxide and lactic acid in the Hanks’ solution, compared to the titanium alloy immersed in the solution with only hydrogen peroxide. According to the crystallization plan (131), TiO_2_ increases in intensity with the immersion of the titanium alloy in the Hanks’ solution and with the addition of lactic acid in the Hanks’ solution, but shows a decrease in the intensity with the addition of hydrogen peroxide and the two compounds in combination (hydrogen peroxide + lactic acid).

The XRD patterns provide information about the crystal structure of the samples observed after immersion in the tested solutions, the formation of titanium dioxide on the surface of the titanium alloy samples, and the absorption of lactic acid on the alloy surface.

The XRD analysis also shows the adsorption of lactic acid on the surface of the alloy from the specific peaks resulting from the immersion of the alloy in Hanks’ solution with LA and the Hanks’ solution with a mixture of lactic acid and hydrogen peroxide. It is also observed that H_2_O_2_ causes a process of destruction of the passive film formed according to the crystallization planes (211) and (111). Its preferential restoration is mainly due to the crystallization plane (131) finally disrupting the formation of the passive film and inducing an increase in the reactivity of the alloy in the respective corrosive environments.

## 4. Conclusions

The study highlights the effects of time and the additions of lactic acid as a metabolic product, hydrogen peroxide as an inflammatory product, and these two in a mixed combination on the reactivity, corrosion behavior, and stability of titanium alloy implant materials through electrochemical methods, SEM-EDX surface analysis, and X-ray diffraction patterns.

The study elucidates the mechanism and kinetics of titanium alloy implants’ degradation in the harsh conditions of inflammation and the presence of lactic acid acting as a metabolic product, which highlights critical issues regarding the corrosion resistance of the studied implant alloy.

The free potential of the titanium alloy immersed in modified biological solutions is affected by adding these compounds to Hanks’ biological solution, which tended create more positive values.

The electrochemical impedance spectroscopy results obtained at immersion time and at different periods after initial immersion time for 168 h showed a slow decrease of the specific resistance due to the addition of the inflammatory product, which causes a decrease in the stability of the oxide film formation and further attacks the corrosion.

A larger decrease of the specific resistance was observed when the titanium alloy was immersed in the to the Hanks’ biological solutions with mixed added compounds as the adsorbed lactic acid inhibited the formation of a passive film.

The results obtained through electrochemical impedance spectroscopy measurements were confirmed by the SEM-EDX analysis and X-ray diffraction patterns. The evident degradation by corrosion was observed when the titanium alloy was in contact with the mixed lactic acid and hydrogen peroxide that were added to the Hanks’ biological solution.

The perturbation of titanium dioxide formation and lactic acid adsorption is proved by XRD.

Overall, the experimental results show that a slow degradation of an alloy implant can occur from an inflammatory situation. A higher level of degradation can occur to the alloy implants under the synergistic effects of multiple compounds—such as lactic acid and hydrogen peroxide—being added to a biological solution—such as Hanks’ solution. The lifespan of a titanium alloy implant can be improved by avoiding these kinds of damaging situations.

## Figures and Tables

**Table 1:** The physical–chemical characteristics of biological Hanks’ solution and modified Hanks’ solution with hydrogen peroxide and lactic acid.

Nr. Crt.	Solution Type	pH	Conductivity (mS/cm)	Salinity (ppt)
1.	Hanks’	7.4 ± 0.5	14.6 ± 0.1	8.8 ± 0.1
2.	Hanks’ + 10 g/L Lactic Acid	1.98 ± 0.1	15.6 ± 0.2	8.9 ± 0.1
3.	Hanks’ + 5 g/L H_2_O_2_	7 ± 0.3	14.8 ± 0.1	8.5 ± 0.1
4.	Hanks’ + 10 g/L Lactic Acid + 5 g/L H_2_O_2_	2.37 ± 0.2	15.2 ± 0.1	8.8 ± 0.1

**Table 2:** The electrochemical parameters of EIS obtained from fitted results.

Solutions	R_s_ (ohm)	R_1_ (kohm cm^2^)	CPE_1_ (µF/cm^2^)	α_1_	R_2_ (kohm cm^2^)	CPE_2_ (µF/cm^2^)	α_2_	Specific R (kohm cm^2^)
Hanks’ t = 0 h	75.35 ± 0.20	13,151 ± 121	86.253 ± 0.18	0.86 ± 0.07	4.885 × 10^7^ ± 0.84	8.65 ± 0.02	0.80 ± 0.01	71,851 ± 64
Hanks’ t = 48 h	83.4 ± 0.15	9000 ± 23	89 ± 0.16	0.86 ± 0.01	7.05 × 10^7^ ± 0.31	0.1 ± 0.0001	0.87 ± 0.02	79,500 ± 124
Hanks’ t = 120 h	70.4 ± 0.22	9000 ± 26	89 ± 0.16	0.85 ± 0.05	7.05 × 10^7^ ± 0.19	1.8 ± 0.01	0.90 ± 0.01	79,500 ± 131
Hanks’ t = 144 h	78.35 ± 0.11	9000 ± 17	82 ± 0.12	0.86 ± 0.01	7.6485 × 10^7^ ± 0.48	6.5 ± 0.06	0.87 ± 0.01	74,000 ± 95
Hanks’ t = 168 h	72.5 ± 0.16	9000 ± 11	94.149 ± 0.21	0.86 ± 0.03	7.822 × 10^7^ ± 0.76	1.8 ± 0.01	0.87 ± 0.01	87,220 ± 101
Hanks’–Lactic Acid, t = 0 h	77 ± 0.39	10 ± 0.1	150 ± 1.1	0.88 ± 0.01	3.05 × 10^8^ ± 0.14	11 ± 0.41	0.98 ± 0.04	305,000 ± 293
Hanks’–Lactic Acid, t = 48 h	77 ± 0.11	10 ± 0.6	150 ± 0.9	0.88 ± 0.07	3.05 × 10^8^ ± 0.22	11 ± 0.37	0.98 ± 0.01	305,000 ± 285
Hanks’–Lactic Acid t = 120 h	77 ± 0.54	10 ± 1.1	150 ± 1.3	0.88 ± 0.01	3.05 × 10^8^ ± 0.18	11 ± 0.23	0.98 ± 0.06	305,000 ± 345
Hanks’–Lactic Acid, t = 144 h	65 ± 0.44	1000 ± 32	48 ± 0.31	0.84 ± 0.05	1.95 × 10^7^ ± 0.95	11 ± 0.32	0.80 ± 0.01	19,500 ± 132
Hanks’–Lactic Acid, t = 168 h	65 ± 0.42	1000 ± 18	48 ± 0.36	0.86 ± 0.01	1.9 × 10^7^ ± 0.14	11 ± 0.15	0.86 ± 0.03	19,000 ± 104
Hanks’–H_2_O_2_ t = 0 h	68.6 ± 0.61	10 ± 0.2	159.1 ± 2.3	0.9 ± 0.08	95,200 ± 74	8.8 ± 0.63	0.80 ± 0.01	95,210 ± 118
Hanks’–H_2_O_2_ t = 48 h	76 ± 0.14	10 ± 0.1	169.1 ± 2.6	0.93 ± 0.03	390,000 ± 35	8.8 ± 0.17	0.90 ± 0.06	390,010 ± 342
Hanks’–H_2_O_2_ t = 120 h	82.22 ± 0.26	10 ± 0.3	171.8 ± 1.9	0.90 ± 0.05	3.656 × 10^6^ ± 0.61	8.8 ± 0.52	0.90 ± 0.01	3656 ± 84
Hanks’–H_2_O_2_ t = 144 h	83 ± 0.69	10 ± 0.1	172.3 ± 0.9	0.90 ± 0.06	4.2442 × 10^6^ ± 0.50	8.8 ± 0.13	0.90 ± 0.03	4244.3 ± 211
Hanks’–H_2_O_2_ t = 168 h	84.33 ± 0.30	10 ± 0.4	169.6 ± 1.3	0.89 ± 0.01	4.266 × 10^8^ ± 0.92	8.8 ± 0.36	0.89 ± 0.01	426,600 ± 94
Hanks’–LA + H_2_O_2_ t = 0 h	60 ± 0.65	100 ± 1.2	55 ± 0.9	0.87 ± 0.008	67,000 ± 130	1.1 ± 0.01	0.88 ± 0.01	67.100 ± 0.51
Hanks’–LA + H_2_O_2_ t = 48 h	58 ± 0.09	100 ± 1.8	120 ± 1.1	0.92 ± 0.01	67,900 ± 52	9.1 ± 0.09	0.92 ± 0.004	68.000 ± 0.89
Hanks’–LA +H_2_O_2_ t = 120 h	68.68 ± 0.18	100 ± 0.9	186.6 ± 2.3	0.92 ± 0.03	73,400 ± 74	9.1 ± 0.03	0.92 ± 0.05	73.500 ± 1.2
Hanks’–LA + H_2_O_2_ t = 144 h	68.03 ± 0.42	100 ± 1.4	200.8 ± 1.7	0.92 ± 0.01	83,300 ± 102	9.1 ± 0.23	0.92 ± 0.03	83.400 ± 2.6
Hanks’–LA + H_2_O_2_ t = 168 h	68.03 ± 0.29	100 ± 1.1	200.8 ± 2.5	0.92 ± 0.006	100,100 ± 98	9.1 ± 0.74	0.92 ± 0.01	100.200 ± 1.3
